# Gemcitabine with Cisplatin Versus Hepatic Arterial Infusion Pump Chemotherapy for Liver-Confined Unresectable Intrahepatic Cholangiocarcinoma

**DOI:** 10.1245/s10434-023-14409-z

**Published:** 2023-10-09

**Authors:** Stijn Franssen, Jessica J. Holster, Joshua S. Jolissaint, Lynn E. Nooijen, Andrea Cercek, Michael I. D’Angelica, Marjolein Y. V. Homs, Alice C. Wei, Vinod P. Balachandran, Jeffrey A. Drebin, James J. Harding, Nancy E. Kemeny, T. Peter Kingham, Heinz-Josef Klümpen, Bianca Mostert, Rutger-Jan Swijnenburg, Kevin C. Soares, William R. Jarnagin, Bas Groot Koerkamp

**Affiliations:** 1 Department of Surgery, Erasmus MC Cancer Institute, Rotterdam, The Netherlands; 2 Department of Surgery, Memorial Sloan Kettering Cancer Center, New York, NY USA; 3 Department of Medical Oncology, Amsterdam UMC, University of Amsterdam, Amsterdam, The Netherlands; 4 Department of Medicine, Memorial Sloan Kettering Cancer Center, New York, NY USA; 5 Department of Medical Oncology, Erasmus MC Cancer Institute, Rotterdam, The Netherlands; 6 Department of Surgery, Amsterdam UMC, University of Amsterdam, Amsterdam, The Netherlands

## Abstract

**Background:**

A post-hoc analysis of ABC trials included 34 patients with liver-confined unresectable intrahepatic cholangiocarcinoma (iCCA) who received systemic chemotherapy with gemcitabine and cisplatin (gem-cis). The median overall survival (OS) was 16.7 months and the 3-year OS was 2.8%. The aim of this study was to compare patients treated with systemic gem-cis versus hepatic arterial infusion pump (HAIP) chemotherapy for liver-confined unresectable iCCA.

**Methods:**

We retrospectively collected consecutive patients with liver-confined unresectable iCCA who received gem-cis in two centers in the Netherlands to compare with consecutive patients who received HAIP chemotherapy with or without systemic chemotherapy in Memorial Sloan Kettering Cancer Center.

**Results:**

In total, 268 patients with liver-confined unresectable iCCA were included; 76 received gem-cis and 192 received HAIP chemotherapy. In the gem-cis group 42 patients (55.3%) had multifocal disease compared with 141 patients (73.4%) in the HAIP group (*p* = 0.023). Median OS for gem-cis was 11.8 months versus 27.7 months for HAIP chemotherapy (*p* < 0.001). OS at 3 years was 3.5% (95% confidence interval [CI] 0.0–13.6%) in the gem-cis group versus 34.3% (95% CI 28.1–41.8%) in the HAIP chemotherapy group. After adjusting for male gender, performance status, baseline hepatobiliary disease, and multifocal disease, the hazard ratio (HR) for HAIP chemotherapy was 0.27 (95% CI 0.19–0.39).

**Conclusions:**

This study confirmed the results from the ABC trials that survival beyond 3 years is rare for patients with liver-confined unresectable iCCA treated with palliative gem-cis alone. With HAIP chemotherapy, one in three patients was alive at 3 years***.***

## Background

Intrahepatic cholangiocarcinoma (iCCA) is the second most prevalent primary liver cancer after hepatocellular carcinoma. The incidence of iCCA in Western countries is approximately 1 per 100,000 and it is increasing rapidly.^1–3^ The majority of iCCAs present at a locally advanced, unresectable stage with limited treatment options due to its late manifestation.^4,5^ Therefore, most patients are only eligible for palliative systemic treatment.^6^ The median overall survival (OS) of patients with unresectable iCCA is about 5 months when no systemic treatment is administered.^7,8^

Palliative systemic treatment for iCCA is typically investigated in studies including all patients with biliary cancers: cholangiocarcinoma (intrahepatic, perihilar, and distal) and gallbladder cancer. The most common regimen for advanced biliary cancers is the combination of gemcitabine and cisplatin (gem-cis), which offers a small OS benefit over gemcitabine monotherapy (11.7 versus 8.1 months, respectively, HR 0.64, *p* < 0.001).^2^ Patients with advanced iCCA mostly have locally advanced rather than distant metastatic disease.^5,9^ A post-hoc analysis of 34 patients with liver-confined unresectable iCCA, treated with gem-cis in the ABC trials, found a median OS of 16.7 months (95% CI 8.2–20.0) and 3-year OS of 2.8%.^10^ These results are a benchmark for any additional locoregional treatment.

Regional treatments are increasingly used to improve OS in liver-confined unresectable iCCA. The main rationale for locoregional treatment of locally advanced iCCA is that most patients die from progressive disease in the liver with biliary obstruction and liver failure.^11^ Liver-directed therapy via a hepatic arterial infusion pump (HAIP) enables the delivery of high-dose chemotherapy (floxuridine) directly into the liver. A continuous flow of intra-arterial chemotherapy is delivered in the hepatic artery via a surgically implantable subcutaneous pump with a catheter in the gastroduodenal artery (GDA), which is a side branch of the hepatic artery. Floxuridine has a 95% first-pass effect, which allows for a 400-fold intra-tumoral concentration compared with systemic administration, without systemic side effects. Three phase II trials from Memorial Sloan Kettering Cancer Center (MSKCC) showed promising results with response rates over 50% and median OS ranging from 25.0 to 30.8 months.^12–14^

The aim of this study was to compare OS after gem-cis versus hepatic arterial infusion pump (HAIP) chemotherapy with or without systemic chemotherapy in patients with liver-confined unresectable iCCA.

## Methods

### Cohort Selection

The study protocol of this multicenter retrospective study was approved by the Institutional Review Board (IRB) of Erasmus MC Cancer Institute Rotterdam (MEC-2021-0501) prior to data collection and processing. Informed consent was waived by the IRB. Data were retrieved from existing medical records.

All consecutive patients diagnosed with liver-confined unresectable iCCA, confirmed by a biopsy, or determined at a multidisciplinary team meeting, were identified. The patients that were treated with systemic gem-cis in Erasmus MC Cancer Institute (Rotterdam, the Netherlands) and Amsterdam UMC (Amsterdam, the Netherlands), were identified between January 2014 and December 2019. The patients that were treated with HAIP floxuridine in Memorial Sloan Kettering Cancer Center (New York, United States), were identified between January 2000 and December 2019. Patients in both groups were excluded if they had undergone prior liver surgery or had distant metastases at time of diagnosis. Patients with locoregional lymph node metastasis (pathologically proven or based on imaging) were not excluded. In the gem-cis group, patients were excluded if they had received first-line chemotherapeutic regimens other than gem-cis. Patients were followed until death or the date they were lost to follow-up.

The following data were extracted for each patient: demographics, baseline hepatobiliary disease, Eastern Cooperative Oncology Group (ECOG) performance status, prior treatment, drainage, date of diagnosis, tumor distribution, cancer antigen (CA) 19-9 serum level at start of treatment, concurrent systemic treatment administration, and date of death or last follow-up. Second-line systemic treatment data were not collected. Tumor distribution included the presence of locoregional lymph node metastasis (pathologically proven or based on imaging), diameter of the largest tumor, and number of tumors. Subsequent locoregional treatments after gem-cis administration or HAIP chemotherapy were also collected.

### Variable Definitions

Liver-confined unresectable iCCA was defined as disease confined to the liver that is unresectable owing to tumor location and/or multifocal involvement and/or locoregional lymph node metastasis. The primary endpoint OS was defined as the time between date of diagnosis and date of death or last follow-up. Resection rate was defined as the percentage of patients who underwent surgery after initial treatment.

Patients’ performance status and CA 19-9 serum level were those measured at the closest time before the start of initial treatment. Multifocal disease was defined as more than one lesion in the liver on imaging, whether it concerned intrahepatic metastases or satellites surrounding the largest lesion. Locoregional lymph nodes were positive or negative for cancer based on pathology results of excisions and biopsies. Locoregional lymph nodes were considered suspicious for cancer on imaging, as defined by a short axis larger than 10 mm and/or a necrotic center of the lymph node assessed by an expert radiologist.

### Statistical Analysis

Continuous variables are presented as mean with standard deviation (SD) or medians with interquartile range (IQR) or range. Differences were tested with the help of Student’s *t*-test or the Mann-Whitney test, depending on the variable’s distribution. Categorical variables are presented as proportions with corresponding frequencies, and differences were tested with the chi-square (*χ*^2^) or Fisher exact test, whichever was appropriate. Missing values were excluded from analysis.

OS was estimated using the Kaplan–Meier method. Patients lost to follow-up were censored. Cox proportional-hazards (CPH) models were used to assess associations between the primary endpoint OS and several variables, including patient demographics and tumor characteristics. The multivariate analysis used the backward selection regression method. Variables that were statistically significant on univariate analysis (*p* < 0.20) were included in the multivariate model along with known relevant variables. Outcomes are presented as hazard ratio (HR) with 95% confidence intervals (CI). Pre-specified sensitivity analyses were performed for first-line (i.e., without previous systemic chemotherapy) and second-line (i.e., with previous first-line systemic chemotherapy) HAIP chemotherapy. Within the subgroup of patients receiving first-line HAIP chemotherapy, patients with and without concurrent systemic chemotherapy were compared. A sensitivity analysis was also performed by adding prognostic factors from the literature (i.e., tumor diameter, serum level CA 19-9) to the multivariate model.

Analyses were performed with the statistical software program R (R Core team, 2021: version 4.1.0, Vienna, Austria) using the package ‘survival’ and the statistical software program IBM SPSS (IBM Corp. Released 2020. IBM SPSS Statistics for Windows, Version 27.0. Armonk, NY: IBM Corp). All tests were two-tailed and statistical significance was defined as *p* < 0.05.

## Results

**Fig. 1:**
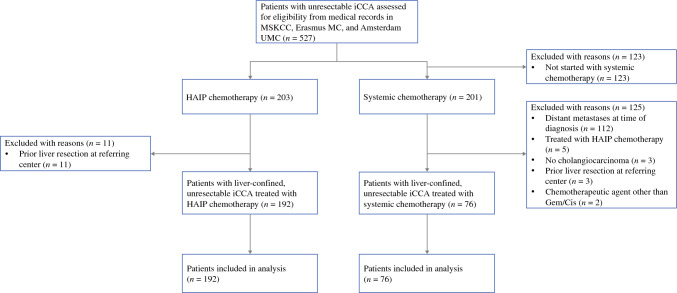
Flow diagram of the inclusion cohort. *iCCA* intrahepatic cholangiocarcinoma; *HAIP* hepatic arterial infusion pump, *MSKCC* Memorial Sloan Kettering Cancer Center, *Gem/Cis* gemcitabine/cisplatin

In total, 268 patients with liver-confined unresectable iCCA were included: 76 in the gem-cis group and 192 in the HAIP chemotherapy group. Of the 76 patients who received gem-cis, 50 were treated in Erasmus MC Cancer Institute and 26 in Amsterdam UMC (Fig. 1). Baseline characteristics are presented in Table 1. In the gem-cis group, 42 patients (55.3%) had multifocal disease compared with 141 patients (73.4%) in the HAIP group (*p* = 0.023). The diameter of the largest tumor was similar in both groups (8.5 vs 8.4 cm, *p* = 0.833). ECOG performance status did not differ between the groups (ECOG 0 or 1, 95.6% vs 97.1%, *p* = 0.214). Baseline hepatobiliary disease was more common in the gem-cis group (18.4% vs 5.2%, *p* = 0.001). Most patients (*n* = 134, 69.8%) received HAIP chemotherapy as first-line treatment; 58 patients (30.2%) in the HAIP group received prior first-line systemic chemotherapy. HAIP chemotherapy was combined with systemic chemotherapy in 138 patients (71.9%), with regimes including gemcitabine with cisplatin or oxaliplatin, gemcitabine monotherapy, and irinotecan (Table 2). Forty-two patients (21.9%) received HAIP as first-line treatment without concurrent systemic treatment.

**Table 1:** Baseline characteristics

Characteristics	Overall cohort (*n* = 268)	Gemcitabine/Cisplatin (*n* = 76)	HAIP, MSKCC (*n* = 192)	*P*-value
	*N* (%)	*N* (%)	*N* (%)	
Female	149 (55.6)	35 (46.1)	114 (59.4)	0.065
Median age, years (IQR)	61.8 (52.4–69.2)	61.2 (52.1–68.4)	61.9 (52.7–69.4)	0.788
ECOG performance status^a,b^				0.214
0	103 (43.2)	36 (52.9)	67 (39.4)	
1	127 (53.4)	29 (42.7)	98 (57.7)	
2	8 (3.4)	3 (4.4)	5 (2.9)	
Baseline hepatobiliary disease				
None	244 (91.0)	62 (81.6)	182 (94.8)	0.001
PSC	5 (1.9)	5 (6.6)	0 (0.0)	
Hepatitis B/C	9 (3.4)	4 (5.3)	5 (2.6)	
Cirrhosis	10 (3.7)	5 (6.6)	5 (2.6)	
CA 19-9, kU/l, (IQR)	77.0 (30.0–408.0)	134 (53.0–1498.0)	64 (30.0–234.8)	0.021
Largest tumor Diameter on imaging, cm, (IQR)	8.5 (5.9–11.0)	8.5 (5.9–10.9)	8.4 (5.9–11.2)	0.833
Multifocal liver disease^c^	183 (68.3)	42 (55.3)	141 (73.4)	0.023
2 or 3 lesions	65 (24.3)	19 (25.0)	46 (24.0)	
4 or more lesions	117 (43.7)	23 (30.3)	94 (49.0)	
Regional lymph nodes^d^	141 (52.6)	39 (51.3)	102 (53.1)	0.890
Biliary drainage prior to start treatment	23 (8.6)	9 (11.8)	14 (7.3)	0.339

*HAIP* hepatic arterial infusion pump, *ECOG* Eastern cooperative oncology group, *IQR* interquartile range.

^a^Before start therapy.

^b^ECOG performance score missing in 30 patients.

^c^Number of lesions missing in 1 patient.

^d^Based on imaging or pathologic confirmation.

**Table 2:** Other locoregional and systemic treatments

Characteristics	Gemcitabine/Cisplatin (*n* = 76)	HAIP, MSKCC (*n* = 192)	*P*-value
	*N* (%)	*N* (%)	
Prior systemic chemotherapy	–	58 (30.2)	
Gemcitabine	–	2 (1.0)	
Gemcitabine/oxaliplatin	–	12 (6.3)	
Gemcitabine/cisplatin	–	29 (15.1)	
Gemcitabine/capecitabine	–	3 (1.6)	
Carboplatin/taxol	–	3 (1.6)	
FOLFIRINOX	–	5 (2.6)	
Other	–	4 (2.1)	
Concurrent systemic chemotherapy	76 (100)	138 (71.9)	
Gemcitabine	–	25 (13.0)	
Gemcitabine/oxaliplatin	–	57 (29.7)	
Gemcitabine/cisplatin	76 (100)	0 (0.0)	
Irinotecan	–	39 (20.3)	
Bevacizumab	–	11 (5.7)	
Other	–	6 (3.1)	
Locoregional treatment^a^	3 (3.9)^b^	18 (9.4)^c^	0.14
Conversion to resection	1 (1.3)	13 (6.8)	0.07
Ablation (RFA, MWA, IRE)	1 (1.3)	2 (1.0)	0.85
TACE	2 (2.6)	3 (1.6)	0.56
External radiation	–	1 (0.5)	0.53

*HAIP* hepatic arterial infusion pump, *Y90* yttrium-90, *RFA* radiofrequency ablation, *MWA* microwave ablation, *IRE* irreversible electroporation, *TACE* transarterial chemoembolization.

^a^Locoregional treatment after gem-cis or HAIP.

^b^One patient was treated with both ablation and TACE.

^c^One patient was treated with both ablation and resection.

### Survival

**Fig. 2:**
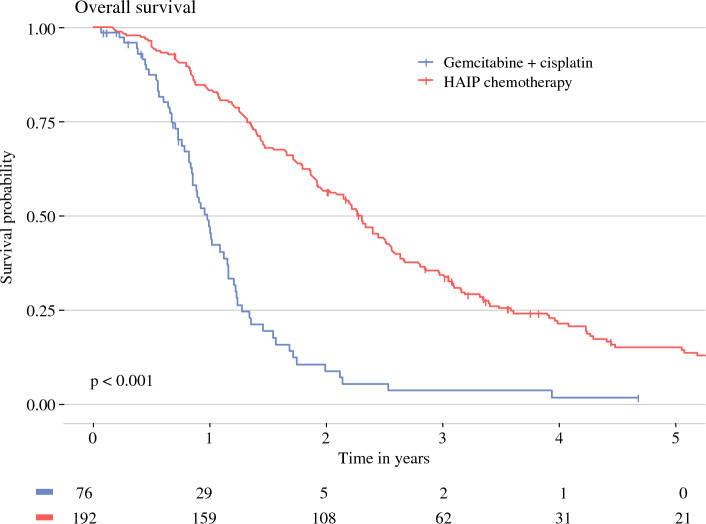
Overall survival (OS) from date of diagnosis. *HAIP* hepatic arterial infusion pump

Median OS for gem-cis was 11.8 months (95% CI 10.3–13.9 months) versus 27.7 months (95% CI 23.7–30.6 months) for HAIP chemotherapy (*p* < 0.001). Three-year OS was 3.5% (95% CI 0.0–13.6%) in the gem-cis group and 34.3% (95% CI 28.1–41.8%) in the HAIP chemotherapy group. Five-year OS was 0% in the gem-cis group and 15.1% (95% CI 10.5–21.8%) in the HAIP chemotherapy group (Fig. 2).

**Fig. 3:**
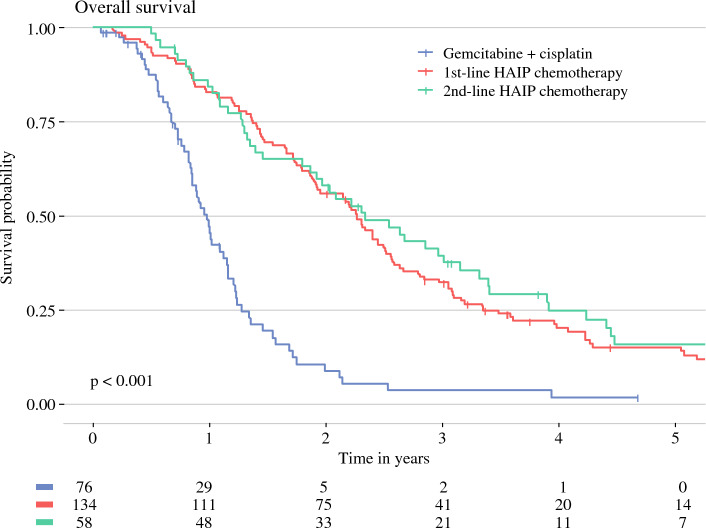
Overall survival (OS) of patients with liver-confined unresectable intrahepatic cholangiocarcinoma (iCCA). *HAIP* hepatic arterial infusion pump

**Fig. 4:**
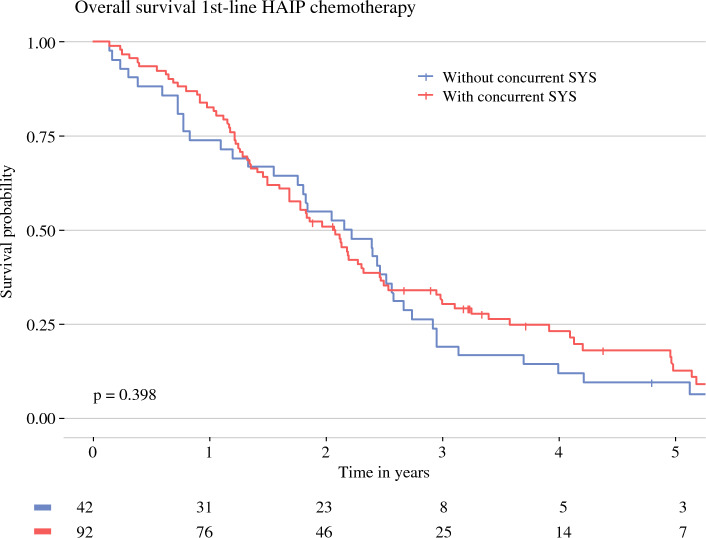
Overall survival (OS) of patients treated with HAIP chemotherapy subdivided by concurrent systemic chemotherapy use. *HAIP* hepatic arterial infusion pump, *SYS* systemic chemotherapy

The subgroup of patients with first-line HAIP chemotherapy had a median OS of 27.2 months (95% CI 23.1–30.3 months) versus 30.0 months (95% CI 23.7–39.8 months) for second-line HAIP chemotherapy. Three-year OS was 32.2% (95% CI 25.1–41.3%) for patients with first-line HAIP chemotherapy versus 39.3% (95% CI 28.4–54.6%) for second-line HAIP chemotherapy (Fig. 3). In the subgroup of patients who received first-line HAIP with concurrent systemic treatment, the median OS was 26.4 months versus 29.4 months for patients without concurrent systemic treatment (*p* = 0.7) (Fig. 4). Three-year OS was 34% (95% CI 25.4–45.3%) for patients who received concurrent systemic treatment and 30% (95% CI 18.7–48.2%) for patients without concurrent systemic treatment.

### Independent Poor Prognostic Factors

Table 3 shows the independent poor prognostic factors including male gender (HR 1.45; 95% CI 1.09–1.94; *p =* 0.01), poor ECOG performance status (2 vs 0, HR 4.22; 95% CI 1.91–9.32; *p* < 0.001), baseline hepatobiliary disease (HR 2.26; 95% CI 1.33–3.84; *p* = 0.003), and multifocal disease (≥ 4 vs 1 lesion, HR 1.51; 95% CI 1.08–2.12; *p =* 0.02). After adjusting for these four poor prognostic factors, HAIP chemotherapy was associated with superior OS compared with systemic chemotherapy alone (HR 0.27; 95% CI 0.19–0.39; *p* < 0.001). After adjusting for additional known poor prognostic factors based on the literature (i.e., tumor diameter ≥ 5 cm, serum level CA 19-9 ≥ 500 kU/l) the adjusted HR for HAIP chemotherapy compared with gem-cis was 0.33 (95% CI 0.22–0.50; *p* < 0.001).

**Table 3:** Univariate and multivariate analysis for overall survival (OS)

Characteristics	*N*	Univariate			Multivariate		
		HR	95% CI	*P*-value	HR	95% CI	*P*-value
Gender	268						
Female		1 [Reference]	–	–	1 [Reference]	–	–
Male		1.44	1.11, 1.87	0.006	1.45	1.09, 1.94	0.01
Age (years)	268	1.01	0.99, 1.02	0.4	NA	NA	NA
ECOG performance status^a^	238						
0		1 [Reference]	–	–	1 [Reference]	–	–
1		1.00	0.76, 1.33	0.98	1.19	0.89, 1.60	0.23
2		2.80	1.29, 6.09	0.009	4.22	1.91, 9.32	<0.001
Baseline hepatobiliary disease	268						
None		1 [Reference]	–	–	1 [Reference]	–	–
Any		2.64	1.69, 4.13	< 0.001	2.26	1.33, 3.84	0.003
CA 19-9 (kU/l)	223						
≤ 500		1 [Reference]	–	–	–	–	–
> 500		1.83	1.31, 2.55	< 0.001	–	–	–
Largest tumor diameter on imaging^b^	253						
≤ 5 cm		1 [Reference]	–	–	–	–	–
> 5 cm		0.98	0.69, 1.40	0.91	–	–	–
Multifocal liver disease	267						
No		1 [Reference]	–	–	1 [Reference]	–	–
2 or 3 lesions		0.89	0.62, 1.28	0.52	0.96	0.65, 1.43	0.85
4 or more lesions		1.32	0.98. 1.79	0.07	1.51	1.08, 2.12	0.02
Distribution	268						
Unilobar		1 [Reference]	–	–	–	–	–
Multilobar		1.01	0.74, 1.39	0.95	–	–	–
Regional nodes^c^	264						
No		1 [Reference]	–	–	–	–	–
Yes		1.21	0.93, 1.58	0.15	–	–	–
Biliary drainage prior to start of treatment	268						
No		1 [Reference]	–	–	NA	NA	NA
Yes		1.03	0.63, 1.69	0.90	NA	NA	NA
Treatment	268						
Systemic chemotherapy		1 [Reference]	–	–	1 [Reference]	–	–
Systemic HAIP		0.27	0.20, 0.37	< 0.001	0.27	0.19, 0.39	< 0.001

*HR* hazard ratio, *CI* confidence interval, *ECOG* Eastern Cooperative oncology group, *PSC* primary sclerosing cholangitis, *HAIP* hepatic arterial infusion therapy, *NA* not assessed.

^a^Before start of therapy.

^b^According to the American Joint Committee on Cancer (AJCC) staging.

^c^Based on imaging or pathologic confirmation.

### Additional Locoregional Treatments

Subsequent locoregional treatment was performed in 3 patients (3.9%) after gem-cis alone compared with 18 patients (9.4%) after HAIP chemotherapy (*p* = 0.14; Table 2). Locoregional treatment mostly involved surgical resection and was performed in 14 patients (5.2%); one patient (1.3%) receiving gem-cis alone and 13 patients (6.8%) receiving HAIP chemotherapy. OS after resection was 61.5% at 3 years and 44.9% at 5 years. Ablation (i.e., radiofrequency ablation, microwave ablation, or irreversible electroporation) was performed in three patients; one patient (1.3%) after gem-cis and two patients (1.0%) after HAIP chemotherapy. Transarterial chemoembolization (TACE) was performed in two patients (2.6%) after gem-cis and 3 patients (1.6%) after HAIP chemotherapy. External radiotherapy was only performed in one patient (0.5%) after HAIP chemotherapy. Selective internal radiation therapy (SIRT) was not performed.

## Discussion

In this study of patients with liver-confined unresectable iCCA, we found that patients had a median OS of 11.8 months when treated with gem-cis alone versus 27.7 months when treated with HAIP chemotherapy with or without concurrent systemic chemotherapy (*p* < 0.001). Three-year OS was 3.5% with gem-cis alone versus 34.3% with HAIP chemotherapy. Within the HAIP group, OS was similar after first-line and second-line HAIP chemotherapy. Moreover, no difference in OS could be demonstrated between patients receiving first-line HAIP chemotherapy with or without concurrent systemic chemotherapy. Independent poor prognostic factors for patients with liver-confined unresectable iCCA were male gender, poor performance status (ECOG 2), baseline hepatobiliary disease, and multifocal disease. HAIP chemotherapy remained an independent favorable prognostic factor (HR 0.33; 95% CI 0.22–0.50; *p* < 0.001) after adjusting for all known confounders.

A SEER population-based cohort study of 5616 patients with advanced intrahepatic cholangiocarcinoma reported a median OS of about 5 months and a 3-year OS of about 3%.^8^ Patients who received gem-cis for liver-confined unresectable iCCA in the ABC trials had a median OS of 16.7 months, with no survivors beyond 3 years.^2^ The median OS after gem-cis in the present study was 11.8 months with a 3-year OS of 3.5%. This median OS was lower compared with the ABC trials, a difference that likely reflects the strict inclusion criteria. In a previous study, we found that about 40% of patients who received gem-cis for advanced iCCA in the “real world” did not fulfill inclusion criteria of the ABC trials.^15^ Regardless of baseline patient characteristics, however, the 3-year OS in any advanced iCCA cohort receiving palliative systemic chemotherapy has been close to zero.

The main rationale for locoregional treatment for advanced iCCA is that most patients die from progressive disease in the liver with biliary obstruction and liver failure. In a large cohort study of 362 patients with iCCA at MD Anderson, the authors found that in patients who were treated with palliative systemic chemotherapy (n = 99), 71 (72%) died from liver failure secondary to local tumor progression.^11^ The impact of local (rather than distant) tumor progression may also explain why the survival curves of patients with liver-confined unresectable iCCA and patients with extrahepatic metastases were overlapping in the subgroup of patients with iCCA who received gem-cis in the ABC trials.^10^

Three phase II trials investigated the combination of systemic and HAIP chemotherapy for locally advanced iCCA.^12–14^ These trials consistently reported a partial response rate of about 50%, a median OS of about 25 months, and a 3-year OS rate of about 35%. A recent systematic review and meta-analysis of HAIP chemotherapy with floxuridine for unresectable iCCA included 154 patients from four studies and found a median OS of 29.0 months (range 25.0–39), a 3-year OS of 39.5% (95% CI 31.5–47.4), and a 5-year OS of 9.7% (95% CI 0.0–23.4).^16^ In particular, the 3-year OS of 35% is an enormous improvement compared with negligible 3-year OS with systemic chemotherapy alone. In our study, most patients (152/192, 79%) who received HAIP chemotherapy also received systemic chemotherapy before or during HAIP chemotherapy. It is unlikely that systemic chemotherapy alone was responsible for the favorable survival outcomes in the HAIP group, considering that 3-year OS was not observed with systemic chemotherapy alone in the ABC trials.^10^ We could not demonstrate a difference in OS between patients who received first-line HAIP with or without systemic treatment. The sample size, however, was low and we believe that HAIP chemotherapy should be given in addition to systemic treatment. HAIP chemotherapy has no systemic toxicity and the complication rates (e.g., biliary sclerosis and pump pocket infection) are low in experienced hands.^17^

Liver-directed therapies are recommended by international guidelines for multifocal or locally advanced (i.e., unresectable) iCCA.^18–21^ iCCA lesions are often too large for percutaneous ablation. Several percutaneous intra-arterial approaches have been investigated, including transarterial chemoembolization and selective internal radiotherapy (SIRT). A phase II trial investigated a combination of first-line SIRT and systemic gem-cis in locally advanced iCCA patients (*n* = 41) with a median OS of 22 months (95% CI 14–52 months).^22^ These results are promising, but appear to be inferior to HAIP chemotherapy. A phase III trial investigating first-line SIRT in patients with liver-confined iCCA was prematurely closed due to lack of accrual (NCT02807181). The main advantage of HAIP chemotherapy compared with SIRT is that lesions are treated across the entire liver, regardless of the number of lesions and whether they are visible on imaging.

This study has several limitations. First, the comparison between the two treatment groups was not randomized and patients were included over a long period of time. We adjusted for known poor prognostic factors, but unknown confounders may have biased the results. For example, about 5% of patients with liver-confined iCCA on imaging have occult peritoneal metastases. These patients would not be eligible for HAIP chemotherapy when metastatic disease is detected at surgical exploration for pump implantation. In the gem-cis cohort these metastases would remain undetected. However, any residual selection bias cannot explain a 3-year OS after HAIP chemotherapy of 1 in 3 patients versus no 3-year OS in the gem-cis group of the ABC trials. Second, data on genomic alterations were missing for most patients, while these alterations are of increasing importance for targeted therapy (i.e., isocitrate dehydrogenase and fibroblast growth factor receptor alterations) and prognosis.^23–29^ In a phase II study, 108 patients with previously treated advanced CCA with FGFR2 fusions or rearrangements received infigratinib. The median OS was only 12 months, but the 3-year OS was about 25%.^25^ In the FOENIX-CCA2 study, 103 patients with iCCA harboring FGFR2 fusion or rearrangements received futibatinib. The median OS was 20.0 months.^28^ In the FIGHT-202 trial, 107 patients with previously treated advanced CCA with FGFR2 fusions or rearrangements received pemigatinib. The median OS was 21.1 months.^29^ As a result of these promising outcomes, three randomized studies in the first-line setting are ongoing (NCT03773302, NCT04093362, NCT03656536). Finally, HAIP chemotherapy is currently offered in only about 60 centers worldwide. It is a complex treatment requiring close collaboration of a multidisciplinary team.

In conclusion, patients with liver-confined unresectable iCCA had a 3-year OS of 34.3% after HAIP chemotherapy compared with 3.5% when treated with systemic chemotherapy alone.
